# Allorecognition by T Lymphocytes and Allograft Rejection

**DOI:** 10.3389/fimmu.2016.00582

**Published:** 2016-12-14

**Authors:** Jose Marino, Joshua Paster, Gilles Benichou

**Affiliations:** ^1^Center for Transplantation Sciences, Department of Surgery, Massachusetts General Hospital, Harvard Medical School, Boston, MA, USA

**Keywords:** allorecognition, T cells, regulatory T cells, allograft rejection, T cell tolerance, major histocompatibility complex, exosomes

## Abstract

Recognition of donor antigens by recipient T cells in secondary lymphoid organs initiates the adaptive inflammatory immune response leading to the rejection of allogeneic transplants. Allospecific T cells become activated through interaction of their T cell receptors with intact allogeneic major histocompatibility complex (MHC) molecules on donor cells (direct pathway) and/or donor peptides presented by self-MHC molecules on recipient antigen-presenting cells (APCs) (indirect pathway). In addition, recent studies show that alloreactive T cells can also be stimulated through recognition of allogeneic MHC molecules displayed on recipient APCs (MHC cross-dressing) after their transfer *via* cell–cell contact or through extracellular vesicles (semi-direct pathway). The specific allorecognition pathway used by T cells is dictated by intrinsic and extrinsic factors to the allograft and can influence the nature and magnitude of the alloresponse and rejection process. Consequently, various organs and tissues such as skin, cornea, and solid organ transplants are recognized differently by pro-inflammatory T cells through these distinct pathways, which may explain why these grafts are rejected in a different fashion. On the other hand, the mechanisms by which anti-inflammatory regulatory T cells (Tregs) recognize alloantigen and promote transplantation tolerance are still unclear. It is likely that thymic Tregs are activated through indirect allorecognition, while peripheral Tregs recognize alloantigens in a direct fashion. As we gain insights into the mechanisms underlying allorecognition by pro-inflammatory and Treg cells, novel strategies are being designed to prevent allograft rejection in the absence of ongoing immunosuppressive drug treatment in patients.

## Introduction

Allorecognition relates to the detection of genetically encoded polymorphisms between individual organisms of the same species by the immune system. Allorecognition has been described in nearly all multicellular phyla, including invertebrates that are devoid of an adaptive immune system (1). Indeed, certain cells of the innate immune system such as NK cells and macrophages are capable of self–non-self discrimination (2, 3). In vertebrates, the adaptive immune response to allogeneic cells is initiated through recognition of polymorphic proteins by T lymphocytes through their antigen receptors. Subsequent activation of pro-inflammatory allospecific T cells initiates a cascade of reactions leading to rejection of transplanted allogeneic tissues and organs. Alternatively, under particular circumstances, deletion or inhibition of alloreactive effector T cells can result in allograft acceptance or tolerance (4, 5). In this article, we review current knowledge of the different pathways underlying alloantigen recognition by different T cells subsets and examine their contributions to rejection or tolerance of allografts.

## Different Mechanisms Involved in T Cell Recognition of Alloantigens

The following section describes the three known pathways (direct, indirect, and semi-direct) by which recipient T cells recognize donor alloantigens [major histocompatibility complex (MHC) and minor antigens] after allotransplantation.

### Direct Allorecognition

Seminal studies in skin-grafted rodents support the view that early after transplantation intra-graft dendritic cells (DCs) (passenger leukocytes) migrate through lymphatics to host regional lymph nodes (LNs) (6, 7). Naïve T cells located in these LNs become activated through recognition of allogeneic MHC molecules displayed on these donor passenger leukocytes (8). This phenomenon, known as direct T cell allorecognition, initiates an inflammatory immune response leading to rapid and acute cellular rejection of skin allografts (9). Unlike conventional T cell responses to nominal protein antigens, the direct T cell alloresponse is polyclonal in that it involves a large portion of the T cell repertoire (1–10%) (10–13). Two non-mutually exclusive mechanisms have been proposed to explain this unique feature of the T cell response against allogeneic MHC molecules: the *high determinant density* and the *multiple binary complex* models (14–16). The high determinant density model postulates that each allogeneic MHC molecule on a foreign cell can be recognized by a single T cell receptor (TCR), which is focused on exposed amino acid polymorphisms of the allogeneic MHC molecule independent of the peptide bound to it. Likewise, various T cells may be activated even if each individual receptor on a given clone displays a low affinity for its ligand. The multiple binary complex model is based on the principle that each individual alloreactive T cell clone interacts with allogeneic MHC molecules bound to a defined peptide. Allo-MHC molecules being occupied by a multitude of different peptides can create many new pMHC complexes that can serve as ligands for various T cell clones. The prevalence of either model in T cell allorecognition presumably depends upon the degree of heterogeneity (structural and/or conformational) between recipient and donor MHC molecules. Unlike conventional immune responses, T cell responses to allogeneic MHC antigens can be observed *in vitro* with T cells isolated from naïve animals cultured with allogeneic irradiated cells. This so-called mixed allogeneic reaction [mixed lymphocyte reactions (MLR)] is believed to rely on the high frequency of precursor T cells capable of recognizing allogeneic MHC molecules. It is also possible, however, that the MLR may reflect the presence of alloreactive memory T cells generated after infections through cross-reactive recognition of self-MHC molecules bound to microbial peptides mimicking an allogeneic MHC–peptide complex, a phenomenon called heterologous immunity (17, 18). For instance, T cells from individuals sensitized to EBV peptides presented by self-MHC class I HLA-B8 also recognize the HLA-B4402 allogeneic MHC molecules (19). Consequently, HLA-B8 individuals display memory T cells directed to HLA-B4402 allogeneic subjects as a result of an EBV infection. The same phenomenon has also been shown in mice after exposure to LCMV and *Leishmania* parasites (17, 20, 21).

### Indirect Allorecognition

Seminal studies by Singer showed that allogeneic MHC class I antigens could be presented by self-MHC class I on antigen-presenting cells (APCs) and trigger the activation of some CD8^+^ cytotoxic T cells *in vitro*, a phenomenon referred to as cross-presentation (22). Most importantly, Lechler and Batchelor provided evidence for an alternative pathway of T cell alloresponse *in vivo* in the early 1980s (23, 24). It was observed that allosensitization could occur in the absence of donor passenger leukocytes following retransplantation of kidney grafts in rats (23, 24). Based on the assumption that donor parenchymal cells were not capable of sensitizing naïve T cells, it was proposed that host MHC class II^+^ bone marrow-derived professional APCs could present alloantigens and initiate an alloresponse. In 1992, our laboratory provided definitive evidence showing that allogeneic MHC peptides were regularly presented by self-MHC class II molecules on recipient APCs and triggered the activation of CD4^+^ T cells in the LNs of skin-grafted mice (25). The relevance of this process, called indirect allorecognition, in solid organ transplantation was documented the same year in two subsequent studies by Fabre and Suciu-Foca’s groups in rats and humans, respectively (26, 27). Subsequent studies documented indirect activation of CD8^+^ T cells after skin transplantation; the relevance of this phenomenon in the rejection process is discussed later in this article (28–30). Determinant mapping and TCR repertoire studies showed that the initial indirect response to an allograft was oligoclonal and followed the rules of immunodominance in that it was mediated by a discrete set of T cell clones directed to a few dominant determinants usually located within polymorphic regions of allogeneic MHC proteins (31, 32). However, progressively, indirect alloresponse by T cells tend to spread to new formerly cryptic allo-MHC peptides (33). Cryptic determinants correspond to peptides that are not processed and/or presented efficiently enough to trigger a T cell response after protein immunization (34). However, T cell responses to these determinants can be elicited upon peptide immunization (34). Secondary responses to formerly cryptic determinants also called antigen spreading has been documented in autoimmune disorders (35, 36) and after allotransplantation and could be involved in chronic rejection (37).

In addition to its role in allo-MHC recognition, indirect T cell recognition is considered as the main driving force being T cell responses to minor antigens (mH), which are peptides usually derived from housekeeping proteins displaying some degree of polymorphism (38). The contributions of mH to the overall indirect alloresponse by T cells and to allograft rejection are discussed later in this article. Finally, it is important to note that it is still unclear where and through which process donor antigens are taken up and processed by recipient APCs and presented to T cells after transplantation. Acquisition of donor antigens by recipient APCs may occur in the graft itself or in the host lymphoid organs through pinocytosis of shed donor proteins, phagocytosis of dead donor cells and apoptotic bodies, or *via* transfer of donor antigens through cell–cell contact or phagocytosis of extracellular vesicles secreted by donor cells.

### Semi-Direct Allorecognition

**Figure 1:**
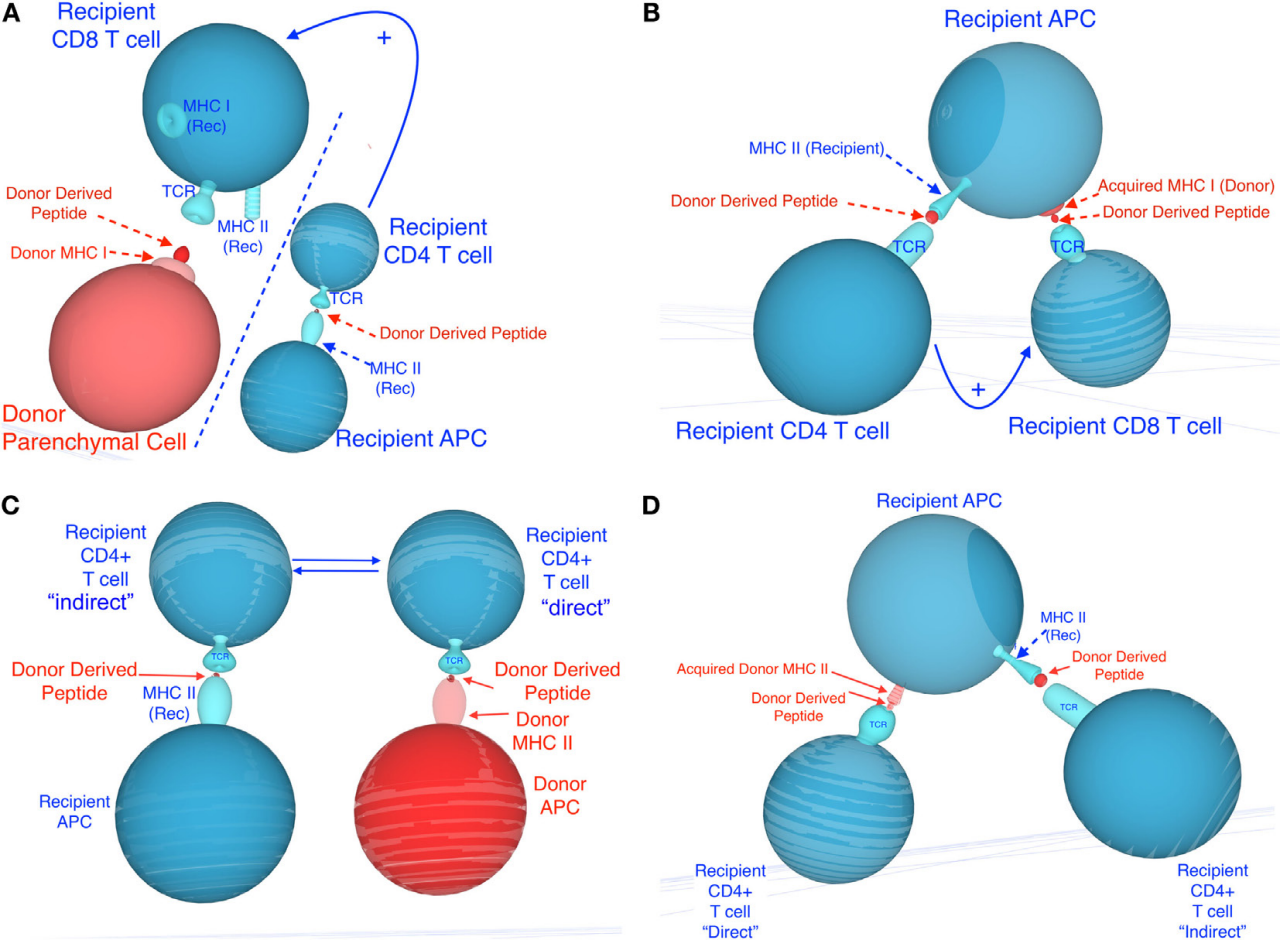
**Potential mechanisms involved in semi-direct allorecognition by recipient CD4^+^ and CD8^+^ T cells**. Recipient cells are shown in blue and donor cells and peptides are shown in red. **(A)** depicts the classical four-cell cluster model in which CD4^+^ T cells activated indirectly [*via* recognition of recipient major histocompatibility complex (MHC) class II^+^ donor peptide, displayed on recipient antigen-presenting cells (APCs)] may provide help for CD8^+^ T cells activated directly (*via* recognition of donor MHC class I on donor APCs). **(C)** depicts the traditional four-cell cluster model in which CD4^+^ T cells activated indirectly (*via* recognition of recipient MHC class II^+^ donor peptide, displayed on recipient APCs) and CD4^+^ T cells activated directly (*via* recognition of donor MHC class II on donor APCs). **(B,D)** describe different mechanisms by which semi-direct allorecognition could allow for a three-cell cluster model in which different T cells recognize different antigens on the same recipient APCs. This process would optimize T cell help by CD4^+^ T cells activated indirectly for CD8^+^ T cell activated directly (recognition of acquired MHC class I) **(B)** and enhance cooperation between CD4^+^ T cells activated directly (*via* recognition of acquired donor MHC class II) and indirectly (recipient MHC class II^+^ donor peptide) **(D)**.

It is now well established that leukocytes exchange molecules, including RNA and proteins, either *via* cell–cell contact (trogocytosis), nanotubes, or through the release of extracellular vesicles such as exosomes (39–41). For instance, T cells were shown to acquire surface immunoglobulin molecules from B cells (42) and antigens from macrophages (43). Likewise, the transfer of MHC molecules between hematopoietic cells was originally documented by Frelinger et al. (44). Acquired peptide–MHC complexes have been shown to remain at the cell surface of APCs for more than 48 h, providing ample opportunities for T cell activation (45). There is accumulating evidence suggesting that this process plays a key role in the initiation and regulation of immunity to microbes and tumors (46). Recent studies have documented the transfer of MHC class I and II molecules (MHC cross-dressing) between recipient and donor DCs after solid organ and bone marrow transplantation (40, 47, 48). At the same time, DCs that have acquired allogeneic MHC proteins *in vitro via* cell–cell contact have been shown to stimulate allospecific T cells *in vitro*, through a mechanism often referred to as *semi-direct allorecognition* (Figure 1) (49–51). It is conceivable that allo-MHC cross-dressing of APCs after transplantation could occur *via* cell–cell contact and through secretion of extracellular vesicles. Lechler et al. have shown that DCs and endothelial cells can acquire MHC complexes *in vitro* and *in vivo* (after DC injections) through cell–cell contact in a temperature- and energy-dependent manner. In these studies, allo-MHC cross-dressed cells induced proliferation of Ag-specific T cells *in vitro* (49–51). On the other hand, a recent study by Marino in our laboratory shows that recipient APCs having acquired donor MHC from donor exosomes trafficking from skin and heart to host lymphoid organs are involved in T cell antigen recognition and activation after allotransplantation. Most exosomes expressed preferentially allogeneic MHC class II and were derived from donor DCs and B cells, i.e., bone marrow-derived professional APCs. However, it is important to note that a significant number of MHC class II^+^ vesicles involved in MHC cross-dressing were not derived from these cells and could potentially be secreted by activated endothelial cells, as suggested by a previous report from Lechler’s laboratory (50). Altogether, these studies involving transfer of MHC antigens provide a different view of the process by which donor passenger leukocyte cells can trigger T cell alloresponses after transplantation. It is now crucial to investigate whether exosomes and allo-MHC cross-dressing are essential elements of the overall alloresponse and allograft rejection processes.

## Relationships Between Different Pathways

Direct and indirect allorecognition represent distinct mechanisms involving different APCs, T cells, and antigen determinants. Each of these pathways can sufficiently and exclusively lead to acute rejection of fully allogeneic skin allografts (52). In certain circumstances, T cells activated directly and indirectly could either cooperate or suppress each other, a process influencing the survival of allografts. It is plausible that in recipients of MHC class I-disparate allografts, CD4^+^ T cells activated exclusively through indirect allorecognition provide help [*via* IL-2 and gamma interferon (γIFN) secretion] for the direct activation of other CD4^+^ T cells (three-cell cluster model) or the differentiation of CD8^+^ cytotoxic T cells recognizing donor MHC class I peptides in a direct fashion (four-cell cluster model) (Figure 1). Likewise, in the absence of bone marrow-derived donor professional APCs, T cells recognizing donor MHC class I or II directly on parenchymal cells can receive costimulatory signals *via* interaction with CD80/86 or CD40 located on recipient professional APCs (activated through indirect presentation to T cells) (trans-costimulation) (Figure 1). At the same time, early inflammatory direct alloresponses associated with γIFN and tumor necrosis factor alpha production and subsequent induction of donor MHC class II expression on endothelial cells presumably enhances allo-MHC antigen processing by recipient APCs and indirect activation of T cells. Therefore, the direct and indirect alloresponses can act synergistically to reject an allograft.

## T Cell Recognition Pathways Involved in Allograft Rejection

Many factors either intrinsic or extrinsic to the graft influence the nature and magnitude of the T cell response induced by a defined pathway of allorecognition. Consequently, the contribution of each T cell allorecognition pathway (direct or indirect) to the rejection process varies upon the nature of the tissue or organ transplanted, the site of the body where it is placed, and the immunological status of the recipient. This section describes some of the factors governing the initiation of direct and indirect alloresponses by CD4^+^ and CD8^+^ pro-inflammatory T cells and the rejection of allogeneic skin, corneal, and heart grafts.

### T Cell Allorecognition in Skin Transplantation

Potent direct and indirect alloresponses by CD4^+^ T cells are induced after transplantation of fully MHC-mismatched skin allografts (13). The direct alloresponse to donor MHC class II antigens by inflammatory CD4^+^ T cells is polyclonal and leads to the rapid rejection of skin allografts (53). Yet, studies from Auchincloss’ laboratory using MHC class II-deficient skin allografts have demonstrated that the CD4^+^ T cell indirect alloresponse was sufficient on its own to cause acute skin graft rejection by providing help for the activation/differentiation of CD8^+^ cytotoxic T cells recognizing donor MHC class I directly (54, 55). This conclusion was further confirmed by experiments using recipient mice adoptively transferred with CD4^+^ T cell clones recognizing donor antigens indirectly (56). In addition, indirect responses by CD8^+^ T cells are also detectable after skin transplantation (28). Studies by Valujskikh and Heeger support the view that indirectly activated CD8^+^ T cells can reject skin allografts following recognition of self-MHC class I^+^ allopeptides present on vascular endothelial cells after replacement of donor graft vessels by recipient ones (30, 57). Therefore, both CD4^+^ and CD8^+^ T cells activated directly and indirectly are elicited after skin grafting and can lead to acute rejection of these allografts. Recent articles by Marino et al. and Smyth et al. support the view that T cells activated through direct and possibly indirect pathway after skin transplantation recognize donor MHC molecules and peptides acquired and displayed by recipient APCs (58, 59). However, the precise contribution of this phenomenon to acute rejection of these grafts remains to be evaluated. Finally, it is important to note that skin allografts that are vascularized at the time of their placement are acutely rejected at the same pace as their conventional (non-primarily vascularized) counterparts, but they do not induce an indirect alloresponse (60). This shows that graft vascularization influences the nature of the allorecognition by T cells after skin transplantation.

### T Cell Allorecognition in Corneal Transplantation

In contrast to skin transplants, corneal allograft rejection is slower and is driven by minor antigens instead of MHC disparities between the host and recipient (61). This unusual feature of corneal transplantation is attributed to the facts that (1) corneal allografts are devoid of MHC class II^+^ APCs at the time of transplantation and (2) they are placed in the eye that is an immune-privileged site of the body (62, 63). These grafts induce indirect but no direct alloresponses by CD4^+^ T cells, a feature presumably associated with the lack of donor MHC class II expression in the cornea (64). In addition, the indirect CD4^+^ T cell alloresponse is directed almost exclusively to minor antigens (61). Such dominance of minor antigens is likely to rely on the low expression of MHC antigens in the cornea [absence of MHC class II and reduced MHC class I expression (65)]. Additionally, in the absence of CD4^+^ T cell direct alloreactivity, indirect alloresponse may be biased toward mH antigens, as observed in the rejection of APC-depleted thyroid grafts (66). On the other hand, CD8^+^ T cells activated directly against donor MHC class I are readily detected after corneal transplantation (67, 68). Although these CD8^+^ T cells produce γIFN, they do not display cytotoxic functions (67, 68).

Only indirectly activated CD4^+^ T cells then drive the rejection process. Interestingly, while no MHC class II^+^ cells were originally detected in the cornea, studies by Dana’s laboratory have documented the presence of DCs in the cervical LNs draining corneal allografts (69). Indeed, CD11c^+^ DCs and CD11b^+^ macrophages are present in the corneal epithelium (70). Interestingly, in “high-risk” recipients of corneal transplants placed in an inflamed eye bed environment (71), corneal DCs express MHC class II molecules as well as CD40, CD80, and CD86 co-receptors at the time of transplantation (71). Consequently, these allografts trigger vigorous direct alloresponses by host CD4^+^ T cells against intact donor MHC class II molecules and are acutely rejected in a few days similar to skin grafts (71). Therefore, lack of immunogenicity of corneal DCs is not an intrinsic property of these cells, but it is due to the microenvironment of the eye. This view is supported by Niederkorn’s studies showing that heterotopic corneal allografts elicit *bona fide* cytotoxic T cell (CTL) responses (72). Likewise, we have shown that corneal allografts placed subcutaneously in mice trigger CD4^+^ T cell direct alloresponses (68). Altogether, these studies demonstrate that both intrinsic (APC contents) and extrinsic (site of placement) factors determine the fate of corneal allografts by influencing the allorecognition pathway and the nature of target alloantigens involved in the T cell response against these grafts.

### T Cell Allorecognition and Rejection of Vascularized Solid Organ Transplants

Early acute rejection of cardiac and kidney allografts is essentially initiated by CD4^+^ T cells recognizing donor MHC class II molecules in a direct fashion (73, 74). These transplants differ from skin allografts in that they are vascularized at the time of their placement (75). This is associated with a rapid trafficking of graft DCs to the host spleen presumably occurring *via* reverse transendothelial vascular migration (76, 77). In addition, some studies suggest that these allografts could be rapidly infiltrated with recipient endogenous alloreactive effector memory T cells (78, 79). These pre-existing memory T cells are present at low frequencies (5–10%) in laboratory rodents (80, 81). In contrast, primates display much higher frequencies (>50%) of alloreactive memory T cells before transplantation (82, 83). These memory T cells may be generated through mimicry with microbial antigens or prior exposure to allogeneic MHC molecules following events such as pregnancy or blood transfusion. We and others have shown that these memory T cells account for resistance to allograft tolerance induction in primates (82–85). Therefore, primarily naïve and presumably endogenous memory T cells activated in a direct fashion mediate early acute rejection of solid organ transplants. Suppression of this response by calcineurin inhibitors and other immunosuppressive agents is regularly achieved in transplanted patients, thereby allowing large-scale clinical transplantation of organs such as kidneys and livers. However, many of these transplants are ultimately lost due to chronic rejection, a process associated with progressive graft tissue fibrosis and blood vessel occlusion (86, 87). There is strong circumstantial evidence suggesting that T cells activated indirectly are responsible for chronic allograft rejection, either on their own or through the induction of alloantibody production by B cells (86–89). The relevance of this concept in clinical transplantation is supported by the detection of donor HLA DR peptide-reactive T cells in kidney-transplanted patients with chronic rejection (90). Additionally, studies by Baker et al. showed the loss of direct and maintenance of indirect alloresponses in renal allograft recipients and its implications in chronic allograft nephropathy in patients (87). Finally, recent studies by Benichou and Morelli’s laboratories suggest that activation of recipient T cells through semi-direct allorecognition might represent an essential element of the immune response to and rejection of cardiac allografts in mice (58, 91). Both studies show that T cells activated *via* this pathway recognized allo-MHC molecules transferred to recipient APCs by donor exosomes released either in the heart transplant or in the recipient’s lymphoid organs (58, 91). Ongoing studies are underway to assess the role of semi-direct alloreactivity in acute and chronic rejection of heart and other solid organ transplants in animal models and patients.

## T Cell Allorecognition Pathways in Regulatory Tolerance

Allograft tolerance, defined as long-term survival of allogeneic transplants in the absence of ongoing immunosuppressive drug treatment, can occur *via* deletion or inhibition of alloreactive T cells. This process can occur naturally, as seen in the tolerance of paternal alloantigens expressed by the fetus during pregnancy (92, 93). In addition, immune-privileged tissues such as the central nervous system and the testis are tolerogenic in that they elicit systemic tolerance to foreign antigens to which they are exposed (94–96). Various cells and mediators of the innate and adaptive immune systems have been implicated in the process of allograft tolerance (4, 96–99). Among them, regulatory T cells (Tregs) play an essential role by suppressing inflammatory responses (100–102). Tregs are CD4^+^CD25^high^ T lymphocytes expressing FoxP3 transcription factor either constitutively (thymic Tregs or tTregs) or after peripheral recognition of antigens (peripheral Tregs or pTregs) (100, 103, 104). In addition to their role in self-antigen tolerance, both Treg subsets can suppress inflammatory alloreactive T cells *in vitro* and *in vivo*. They inhibit alloreactivity in MLR *in vitro* (4, 96, 99, 105) and are thought to mediate transplant tolerance elicited *via* leukocyte costimulation blockade, donor-specific transfusion (4). This is supported by experiments in which inoculation of Tregs from tolerant mice to naïve mice could prolong allograft survival and even transfer tolerance (4). In addition, studies from Colvin’s laboratory using FoxP3-diphtheria toxin receptor mice showed that *in vivo* deletion of Tregs abrogated ongoing tolerance to kidney allografts in mice (106). tTRegs are positively selected in the thymus medulla based on their high affinity for self-antigen pMHC complexes (107). While tTregs require TCR interaction with self-MHC class II molecules to mediate their suppress functions, they are thought to be non-antigen specific. Indeed, tTregs isolated from naïve mice can suppress T cells responding to polyclonal stimulators (anti-CD3/anti-CD28 mAbs or PMA/ionomycin) and MLR regardless of the nature of the allogeneic stimulators. The nature of the self-peptide determinants recognized for tTregs is not known. Studies from LeGuern’s laboratory suggest that tTreg recognition is biased to self-MHC class II peptides bound with self-MHC class II molecules themselves (referred to as Tlo) (108). Tolerance of solid organ transplants in swine and rodents *via* allo-MHC class II transgenesis support this view (109–113). In contrast to tTregs, pTregs presumably acquire FoxP3 expression and suppressor functions through recognition of donor antigens (MHC and/or minor antigens) presented by selected APCs (immature DCs and plasmocytoid DCs) in an appropriate cytokine milieu (4, 114–117). Although activation of pTregs may be antigen specific, it is not clear whether their suppressive function follows the same rules. Therefore, both Treg subsets involved in allograft tolerance are presumably activated through recognition of peptides presented by self-MHC class II on recipient APCs, i.e., in an indirect fashion. However, the mechanisms by which they suppress alloreactive T cells and induce and/or maintain allograft tolerance are still unknown.

## Concluding Remarks

It is now firmly established that the mechanisms by which T cell recognize and respond to alloantigens greatly vary upon the nature of the transplanted organ or tissue, the site of anatomical placement, and the immunological status of the host. This explains why certain transplants, such as skin allografts, which induce potent inflammatory responses by both CD4^+^ and CD8^+^, activated directly and indirectly, are highly immunogenic and thereby resistant to tolerance induction. In contrast, corneal allografts that elicit only indirect alloresponses by CD4^+^ T cells are tolerogenic and often spontaneously accepted. On the other hand, early acute rejection of solid organ allografts such as hearts and kidneys is mediated essentially by T cells activated directly. While this immune response results in a potent inflammatory reaction, it is readily inhibited by calcineurin inhibitors. This explains why these drugs have been effective at achieving prolonged survival of organ allografts in patients. These treatments do not, however, efficiently suppress alloreactive memory T cells, thus precluding transplantation in patients sensitized to their potential donors (10% of patients). Most importantly, many transplanted organs are progressively lost due to chronic rejection, a process presumably initiated by indirectly activated T cells and subsequent production of cytotoxic anti-donor antibodies. For reasons that are still unclear, this response is not always efficiently suppressed by current immunosuppressive drugs. Therefore, future challenges in clinical transplantation will be to suppress or eliminate allospecific memory T cells and to prevent the development of indirect alloresponses.

## Author Contributions

All the authors listed have made substantial, direct, and intellectual contribution to the work and approved it for publication.

## Conflict of Interest Statement

The authors declare that the research was conducted in the absence of any commercial or financial relationships that could be construed as a potential conflict of interest.

## Abbreviations

MHC, major histocompatibility complex; APC, antigen-presenting cell; Treg, regulatory T cell; tTreg, thymic regulatory cell; pTreg, peripheral regulatory T cell; γIFN, gamma interferon; TNFα, tumor necrosis factor alpha; DST, donor-specific transfusion; DTR, diphtheria toxin receptor; TMEM, memory T cell; CTL, cytotoxic T lymphocyte; DC, dendritic cell.
